# The Proposed Royal Commission in Relation to Maternity

**Published:** 1913-09-27

**Authors:** 


### MATERNITY AND MOTHERING.

### The Proposed Royal Commission in Relation to Maternity.

One of the burning questions of the moment, as those who have followed the discussions at the recent International Congress of Medicine and read the preceding article must be fully aware, is the control and, if possible, the abolition of infective venereal diseases by some form of concerted medical action initiated through the State. Should the suggested Royal Commission come into being, the presentation of its report will mark a very important milestone in social progress; and further advance will then depend very largely upon public opinion, without the support of which no politician of either party is likely to proceed to action in the matter.

Of the two important diseases which will form the subject of the Commission's labours (when it comes into existence), both can, and frequently do, affect very seriously the health of mothers and babies; so that their relevance to both maternity and infancy is obvious. It is difficult to be certain which of these two diseases produces the more mischief in these respects. One, gonorrhœa, is responsible for a very considerable proportion of female sterility, and for an equally considerable proportion of pelvic illnesses, operation cases, and even occasional deaths. It is also the cause of about one-third of the cases of blindness from infancy that exist in this country. The other disease, syphilis, is believed to be less common; but its effects are even more disastrous, comprising as they do not merely a long tale of consequential illnesses of many kinds, but also a tendency to repeated miscarriages, to stillbirths, and to highly defective offspring.

Of all these things the ordinary young woman of marriageable age is totally ignorant; and in the majority of cases, fortunately, she remains in that state of bliss all through her experience of marriage, maternity, and mothering. But there is a minority, and by no means a negligible minority, who make painful acquaintance with these terrible diseases, and get to know part, if not the whole, of the truth about them by sad experience. Most frequently the innocent victim is the young wife; but sometimes it is the husband. Again, the contagion may be acquired accidentally, an occurrence by no means so rare—at least in the case of syphilis—as is popularly imagined. At all events, once infection is introduced into a family, however its entrance is effected, the results are so appalling and so disastrous that only those who have witnessed them, as medical men are often called upon to do, can appreciate the full magnitude of the terrible evils which these diseases cause.

It is the duty of every citizen to know that these diseases are very prevalent, and what their effects upon the national health are. It is his duty to digest such information as he can obtain from reliable sources concerning the modern methods of treatment evolved during the last four or five years, and to learn how those methods throw an entirely fresh light on the problem.

This duty we cannot doubt that the citizens of the day will shoulder, if once they are properly instructed by their expert advisers—the medical profession. If they do not, if they go on in undisturbed complacency in spite of an energetic educational campaign, there is no doubt that the ardent feminists will take the matter up, nor shall we blame them if they do. For reasons which seem to the writer convincing, though they cannot be set down here, this problem is one which men are best fitted to cope with. Meanwhile he would point out that the facts above stated as to the relations of venereal disease to maternity and to infancy concern vitally every man in the kingdom, married or unmarried. And if anyone is unwilling to believe this, let him read a letter in the correspondence columns of the *Daily Mail* for August 16 (one of the most pitiful human documents it is possible to conceive), or Brieux plays for that matter.

In the above an attempt has been made to avoid elaboration of details, partly because it is so easy for the non-medical public to misunderstand medical terms and because it is essential that an atmosphere of groundless suspicion and mistrust should not be created in those homes, the great majority in fact, where there is no shadow of reason for it. Yet in Dr. R. W. Johnstone's recently published Report to the Local Government Board it is stated that venereal infections account for from 25 to 50 per cent. of the operations at women's hospitals. Admitting fully the serious total of the operations thus necessitated, the writer believes, after several years' practice in a women's hospital, this estimate to be too high.

